# Rejoinder to a Reply to a Review of Dr. Eve’s Contribution on the History of Hip-joint Operations

**Published:** 1868-08

**Authors:** George A. Otis

**Affiliations:** Ass’t Surgeon and Brev’t Lt. Col. U. S. Army


### Correspondence.

### Rejoinder to a Reply to a Review of Dr. Eve's Contribution on the History of Hip-joint Operations.

*Mr. Editor:*—My attention has been recently called to a review of Prof. Eve's statistical paper on hip-joint operations, in the January number of the *Buffalo Medical and Surgical Journal*, and to a reply to that review by Dr. Eve in the February number of the same journal. As both the reviewer and the professor allude to a report of mine on the same subject, I respectfully ask to be heard also.

In his paper published in the *Transactions of the American Medical Association*, vol. xviii, p. 271, Dr. Eve states that he reports twenty cases of coxo-femoral amputation "performed during the late civil war in the Southern service," that he knows "positively that these were not all the cases that were operated upon during the war in the Southern States," but thinks it probable that his statistics embrace a pretty accurate account "of all the successful cases, at least;" he does not think a question can arise as to the success of four in the twenty cases; and, after admitting that he reports all of the successes but not all of the reverses, he claims a "success of four in twenty cases, or one in five." He finds the mortality after coxo-femoral amputation for injury in the U. S. Armies to be "precisely double what statistics make it to have been in the Southern service." He gives a table of thirteen resections of the hip-joint for injury, and decides that "five may be considered successful," while he sets down the fatality of this operation in the United States service as "nearly four times greater than on the Southern side," and states that his investigations have "led the searcher unwittingly to a most favorable result on the side least expected."

The reviewer in the *Buffalo Journal* observes that Dr. Eve proceeds with his comparison "rather exultingly;" regrets that so distinguished a surgeon should have hazarded such premature conclusions—declares that the evidence adduced of the success of some of the cases is insufficient, and expresses the belief that a careful study of the operations for excision of the head of the femur will prove that "no such disparaging difference will be found to exist as the investigations of Prof. Eve would make appear."

It is believed that very few readers except Dr. Eve will deem these strictures harsh or intemperate. Dr. Eve, however, feels called on to reply to them. In the February number of the Journal, he asserts that the criticisms on his paper "emanate from the old sectional feeling;" that his investigations were made "at the special request and for the use of the Surgeon-General of the U. S. Army;" that "re-amputations must not be counted," and that "courtesy requires that when even a doubt is thrown on an author's statements, the editor should at least notify him of it, that he may have space in the same paper for defence."

Professing not only to cast doubt upon many of Prof. Eve's statements, but to refute them, I have sent to him a copy of this paper.

It is perfectly true that on July 11th, 1866, having been informed by Dr. O. A. White, formerly surgeon P. A. C. S., that Professor Eve had two or three times performed amputation at the hip-joint for gun-shot injury, I wrote to Dr. Eve as follows: "I shall be greatly indebted to you for information in regard to the cases in which you operated, or for any cases in the Confederate army which came to your knowledge," and that subsequently Professor Eve communicated to me memoranda of twenty cases of amputation at the hip-joint, sixteen of which were new to me. Of most of these cases I procured additional details from the operators or from other sources; but I made the fullest acknowledgment of my indebtedness to Dr. Eve in the report published in *Circular No.* 7, S. G. O., July, 1867. In my correspondence with Dr. Eve, I wrote personally; but unquestionably he regarded the data contributed as designed for the official use of the Medical Bureau, and so expressed himself in his correspondence with Drs. Gilmore, Compton and others, from whom he solicited reports, and I accordingly returned thanks to him in the name of the Surgeon-General. How far, under these circumstances, the courtesies of authorship permitted Dr. Eve to publish these materials in the *Transactions of the Medical Society of Tennessee* and the *Transactions of the American Medical Association*, prior to the official publication of the bureau for which they were ostensibly collected, I shall not presume to judge.

I now invite attention to the following facts in regard to amputations at the hip-joint for gun-shot injury by medical officers of the U. S. Army:

JAMES E. KELLY, a survivor of primary amputation, resides at Black Lick, Indiana county, Pennsylvania, and was in good health in April, 1868, when he went to New York and had an artificial limb adjusted by Dr. E. D. Hudson.

GEORGE W. LEMON, who had survived a secondary coxo-femoral amputation nearly four years, was examined by the writer at Dr. E. D. Hudson's office in New York City, on March 31st, 1868. He was able to walk very well with an artificial limb and cane.

WOODFORD LONGMORE, amputated secondarily at the hip-joint January 18th, 1866, has recently been reported by Dr. G. C. Blackman as in good health. He lives near Cincinnati, Ohio.

EBEN E. SMITH, re-amputated at the hip January 19th, 1865, was an inmate of the Soldiers' Asylum, near Augusta, Maine, and is in good health. In May, 1868, he visited New York to procure a prothetic apparatus.

EDWIN D. ULMER, who underwent a re-amputation at the coxo-femoral articulation, February 17th, 1866, is in vigorous health, is engaged in business in a publishing house in Philadelphia, and wears a limb made by Clement.

LEWIS FRANCIS, re-amputated at the hip, May 21st, 1864, is an old and rather dissipated man. He is now in poor health, having, probably, necrosis of the os pubis. He resides in Hamilton street, Brooklyn, N. Y.

There are living, at the present day, to be identified by whosoever chooses to take the trouble, the survivors of one primary, two secondary, and three re-amputations at the hip-joint for gun-shot injury, performed during or after the late war by U. S. surgeons. The entire number of cases of this operation performed by medical officers of the U. S. Armies and recorded in the Surgeon-General's office is 33. An unsuccessful primary operation has been reported to the Surgeon-General's office also by Surgeon Gorgas, U. S. Navy. The percentage of mortality in these thirty-four operations is therefore 82.35. If, as Prof. Eve maintains, the re-amputations should be deducted, we will not set aside, as he does, the successful re-amputations only, but the successful and fatal alike. This leaves 28 operations with three well attested cures, not 34 cases and only 3 successes, as Dr. Eve ingenuously states.

Examining now the twenty cases of coxo-femoral amputation as "performed during the late civil war in the Southern service," we find that Dr. East's case (*Southern Journal Medical Sciences*, vol. 11, p. 232) was that of a negro wounded in a quarrel with another negro in Western Texas, in the latter part of 1864, the operation being performed ten hours after the comminution of the head of the right femur by a rifle ball. Dr. East had "mislaid the notes" and reported the case from memory two years subsequently. Dr. Douglass, a son-in-law of the master of the negro had told Dr. East, at Vicksburg, in the winter of 1865, that the negro had recovered, and was still with his master in Lavacca county, Texas. The report by Dr. East does not inspire confidence by its precision. It is not surprising that the profession should exact incontrovertible evidence of a successful primary coxo-femoral amputation for gun-shot injury, an achievement which has been regarded for the last ten years as almost hopeless.

The next case about the success of which Prof. Eve thinks no question can arise is that of private Williamson, 13th Mississippi regiment, amputated at the hip, June 4, 1862, by Dr. J. T. Gilmore, of Mobile, Alabama. This patient went home with his wound cicatrized six weeks after the operation. The most diligent inquiries have failed to trace his ulterior history. Dr. Eve chooses to regard this as an unequivocal success; but the modest and conscientious operator, remembers that the patients of M. Legouest and Dr. Weir died three and four months after operation, and advances no such claim.

The third alleged success is that of Dr. W. M. Compton, of Holly Springs, Miss., in the case of private Robinson, of a Louisiana regiment, amputated at the hip primarily at Fort Pemberton, March 13, 1863. He left the Yazoo City hospital to go to his home April 20, 1863. Dr. Eve states that he "was well a year afterwards;" but the latest report from him is given by Dr. J. M. Green, of Aberdeen, Miss., who *heard* that the patient was in good condition in August, 1863, less than *six months* after the operation. Every effort has been unavailingly made by the medical officers stationed in Louisiana to trace the ultimate history of the patient. He cannot be found.

The fourth case reported as successful was so undoubtedly, and was reported by the operator, Dr. A. M. Fauntleroy, in the first number of the *Richmond Medical Journal*, January, 1866. As it was a *re-amputation*, it is somewhat singular that Professor Eve retains it in his comparative estimate! For does he not discard the successful re-amputations of the Northern surgeons?

Setting aside Dr. East's case, which, whether successful or otherwise, was certainly not "performed in the Southern service," we have 19 cases, 1 success, and 2 doubtful and possibly successful cases. Eliminating Dr. Fauntleroy's operation of re-amputation, as Dr. Eve says, "every statistician knows is right and proper," we have 18 cases, and 2 possible successes, or 1 success in 9 operations. It has been shown above that in the United States armies there were 3 perfectly attested successes in 28 operations, or about 1 in 9, and so we arrive at the result that would be anticipated by every one whose scientific perceptions were not obscured by partisan prejudices, that the mortality after great operations in the Union and rebel armies, when the patients were placed under similar conditions, was about equal.

Following Dr. Eve's statistical method, however, we might evolve a very different result. Taking the twenty cases reported by him, and the unsuccessful cases which he mentions, but does not tabulate, by Drs. Nott, Hinkley, Cowan and Crymes, and two fatal operations by Dr. Benjamin D. Lay, which have been reported to the Surgeon-General's office, in addition to the one recorded by Dr. Eve, we should have a total of 26 operations with 1 well attested recovery, (which was a re-amputation) 3 doubtful cases, and 22 deaths, or 1 success in 23 cases. But this would be imitating the numerical inaccuracies we condemn, and would be particularly unfair, inasmuch as it is now perfectly well ascertained that neither Dr. Nott nor Dr. Hinkley ever performed amputation at the hip-joint for gun-shot injury.

Of the 13 "resections of the hip-joint" for injury tabulated by Prof. Eve, the first two are authentic and were reported in the *Confederate States Medical and Surgical Journal*, vol. 1, p. 5, January, 1864. The first, a successful case, was included in the table in *Circular, No.* 6, S. G. O., 1865. (Case xiv, p. 66) with which Dr. Eve compares his statistics. It assuredly cannot be used on both sides in a comparative estimate. The third case, that of Humphrey, Co. H, 1st Virginia Infantry, was investigated by the Surgeon-General's Office long belong the publication of *Circular No.* 6. The patient was under the charge of Surgeon H. S. Hewit, the senior surgeon of the U. S. Volunteer staff, who declares that no operation was performed while the patient was in hospital at Frederick. The man was subsequently transferred to Baltimore, and was under the charge of Prof. Miltenberger, who, in a letter to the Surgeon-General's Office, dated March 20, 1868, states that no secondary operation was performed there. The case was nothing else than a consolidated gun-shot fracture of the trochanteric region. The tenth case is also pronounced successful, and is ascribed to Assistant Surgeon Maurice J. Asch, U. S. A., on the authority of Dr. D. D. Saunders, of Memphis, Tennessee, (*Memphis Medical and Surgical Monthly*, vol. 1, p. 81,) who states that he saw the patient eighteen months subsequent to the operation, which was performed after the battle of Gettysburg. But Dr. Asch informs the writer that he did not excise the head of the femur after Gettysburg, and, indeed, that he has never performed that operation. He did treat after Gettysburg, a confederate soldier, private Durbin, 9th Alabama regiment, with a gun-shot fracture in the upper part of the shaft of the femur by extracting some detached fragments of bone, and the man recovered and was exchanged, and was possibly the patient seen by Dr. Saunders. But even if Dr. Asch's patient and that of Dr. Hewit had recovered after excision of the head of the femur, it is difficult to understand how credit should thereby accrue to Southern surgeons. The successful case ascribed to Dr. Avent is reported as follows: "An officer * * * gun-shot fracture of neck of femur; wound dilated at entrance and spiculæ removed with saw and forceps; lingered for months, but finally recovered with a limb four inches short." Of which it may be said, that nothing indicates that either the projectile or the surgeon implicated either the head of the femur or the hip-joint. Of Dr. Ladd's case it is recorded that it was "considered successful," though the patient survived only sixty days.

It appears then that of the thirteen alleged examples of resection at the hip-joint, two cases, (Miltenberger's and Asche's) claimed as successful, are proved not to have been examples of the operation; and that the proof in regard to a third alleged successful case (Avent's) is quite insufficient. The table is thus reduced to ten cases, with one successful result; for we take it for granted that no statistician but Dr. Eve will admit the case of Dr. Ladd, which terminated fatally in sixty days, as a success, however it might have been "considered" by the operator.

It is related by M. Guizot, that on the occasion of some quarrel at the French Institute, the Emperor Napoleon I, said one day to M. de Fontanes, with somewhat disdainful irony, "leave us at least the republic of letters." One might hope also that the field of science should not be invaded by partisan prejudice. The mortality and the merits of the operation of excision of the head of the femur for gun-shot injury, will be hereafter judged, in a large measure, by statistics derived from the experience of surgeons of both Northern and Southern armies during the war of the rebellion, though compiled in a different manner from those of Dr. Eve. But it will remain a matter of record that that distinguished Professor was the first to institute comparisons of the results obtained by surgeons of the two armies, comparisons which must be pronounced hasty and premature and inaccurate, even if not considered invidious, and that the tendency of such comparisons was to intrude into surgical literature contentions that should be foreign to that arena.

Very respectfully, your obedient servant,

George A. Otis,

Ass't Surgeon and Brev't Lt. Col. U. S. Army.

